# A Systematic Examination of Colour Development in Synthetic Ultramarine According to Historical Methods

**DOI:** 10.1371/journal.pone.0050364

**Published:** 2013-02-13

**Authors:** Ian Hamerton, Lauren Tedaldi, Nicholas Eastaugh

**Affiliations:** 1 Department of Chemistry, Faculty of Engineering and Physical Sciences, University of Surrey, Guildford, Surrey, United Kingdom; 2 Art, Access and Research, London, United Kingdom; Queen's University Belfast, United Kingdom

## Abstract

A number of historical texts are investigated to ascertain the optimum conditions for the preparation of synthetic ultramarine, using preparative methods that would have been available to alchemists and colour chemists of the nineteenth century. The effect of varying the proportion of sulphur in the starting material on the colour of the final product is investigated. The optimum preparation involves heating a homogenised, pelletised mixture of kaolin (100 parts), sodium carbonate (100 parts), bitumen emulsion (or any ‘sticky’ carbon source) (12 parts) and sulphur (60 parts) at 750°C for *ca.* 4 hours. At this stage the ingress of air should be limited. The sample is allowed to cool in the furnace to 500°C, the ingress of air is permitted and additional sulphur (30 parts) is introduced before a second calcination step is undertaken at 500°C for two hours. The products obtained from the optimum synthesis have CIE ranges of *x*  = 0.2945-0.3125, *y*  = 0.2219–0.2617, *Y*  = 0.4257−0.4836, L* = 3.8455–4.3682, a*  = 4.2763–7.6943, b* = −7.6772–(−)3.3033, *L*  = 3.8455–4.3682, *C* = 5.3964–10.8693, *h* = 315.0636–322.2562. The values are calculated using UV/visible near infrared spectra using Lazurite [1], under D65 illumination, and the 1931 2° observer.

## Introduction

The colour blue crops up in art through history in the works of great artists, sculptures and ancient civilisations and a fast, vivid blue was sought by artists. Yves Klein, who started using only blue for his paintings and sculptures, stated, *‘Blue has no dimension, it exceeds everything…All colours evoke associations…, whereas blue is reminiscent of the sea and the sky, which are the most abstract parts of the tangible and visible nature* [1]. Historically, the ancient Egyptians produced an important pigment now widely known as Egyptian Blue (C.I. Pigment Blue 31); it was laborious to produce, utilised expensive materials and much skill was required to create it, which explains why the pigment was only used in tombs of the wealthy. Lazurite (or natural ultramarine) is arguably the most expensive source of blue pigment. Formed from the ground, semi-precious stone *lapis lazuli* (found in treacherous mountain locations), Lazurite was exceedingly expensive, costing up to 11000 French Francs per kilogramme FrF/kg [2]. Despite these drawbacks, Lazurite was undoubtedly the most favoured blue colour: the pigment worked well in water and oil, did not fade, and gave a very consistent shade. So highly prized was the beauty of the blue derived from *lapis lazuli*, that it excited much comment and historical texts are littered with attempts to recreate artificial lazurite as ‘ultramarine’ and in the 19^th^ century, alternative synthetic blue pigments, such as cobalt blue, cobalt aluminium oxide (CoO.Al_2_O_3_) and Prussian blue (iron(III) hexacyanoferrate(II)), were developed. However, the colours were inconsistent and poor quality blue pigments like smalt, a cobalt doped glass, could actually damage the appearance of the canvas. Cheaper alternatives such as azurite have also been employed and the expense and preparation of the blue pigment used can often inform one of the importance and standing of the artist and benefactor. For example, occasionally, in a depiction of Christianity, most of the blue will be painted in a lesser pigment, such as azurite, whilst the robes of Mary and sometimes Peter, will be depicted with ultramarine, showing respect for the figures and the religiosity of the time in which the work was painted [3].

**Figure 1:**
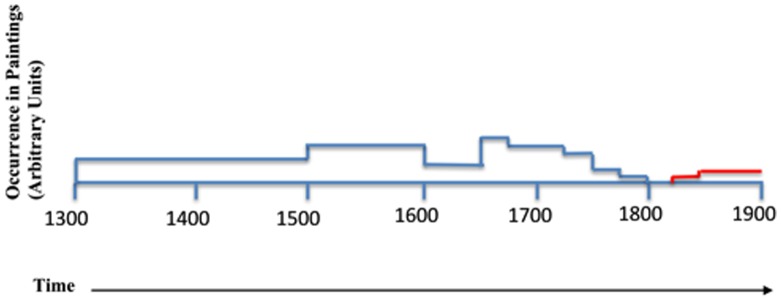
Use of natural ultramarine (blue) and synthetic ultramarine (red) amongst paintings in the Schack-Galerie, Munich, from reference 4.

In 1824 the French chemist Vacquelin communicated to *La Société d’Encouragement pour l’Industrie Nationale*, that a by-product of glass manufacture produced a structurally similar product to natural ultramarine in soda kilns. Consequently, *La Société* responded by offering a 6000 FrF prize to anyone who discovered an economic industrial process whereby synthetic ultramarine could be produced for less than 300 FrF/kg. After some controversy in which the noted German chemist, Gmelin, claimed he deserved both the financial and academic reward, the prize was eventually awarded to the Frenchman Guimet, in 1828 [2]. The French secret method produced synthetic ultramarine at a mere 850 FrF/kg, making the pigment significantly more affordable and widening the market for the colour. Kuhn documented the nature of ultramarines pigments used in paintings exhibited within the Schack-Gallerie in Munich [4] and the general trends in the use of ultramarine can be seen to shift through the centuries (Figure 1); predictably, the drop-off in the use of natural ultramarine is largely attributed to the introduction of the cheaper, synthetic alternative. The use of natural ultramarine, pre-1824 is not uniform as trends developed depending on artists’ recommendations and the availability of the mineral. For example, in the 16^th^ century a growth in the use of natural ultramarine is seen, coinciding with a recommendation from a prominent miniaturist, Nicholas Hilliard [2]. Naturally, miniaturists could afford to be somewhat more flamboyant with their choice of pigments. As the explosion of cheaper blue pigments took hold, so artists’ palettes began to develop. For instance, synthetic ultramarine is used in Pissarro's *The Côte des Bouefs,* Monet's *Gare Saint-Lazare* and perhaps most strikingly and effectively in Renoir’s *Les Parapluies*.

Precise and succinct historical descriptions of pigment synthesis are rare and this is particularly true of ultramarine. The first synthetic preparation of ultramarine was shrouded in secrecy, and although attempts to reproduce it have been carried out ever since, success rates have varied considerably. This is largely due to the fact that ‘recipes’ for the pigment appear to always leave out key pieces of information, such as specific temperatures and optimum times. To compound matters, texts often discuss, at length, features of the synthesis that are peculiar to a particular person or industry, and are by no means essential. The aim of the current work was to examine several prominent historical treatises describing the preparation of ultramarine using spectroscopic methods, identify and evaluate the essential, desired and optimal factors for the synthesis of this time-honoured, revered, and expensive pigment.

## Experimental

### Apparatus

The furnace used was a Carbolite CWF1100 (alumina crucibles were purchased from Fisher). A Specac pelletizer was used to produce solid pellets. Ultra-violet/Visible spectra were obtained using a Perkin Elmer Lambda 750 UV-vis-NIR spectrometer. Owing to the solid-state nature of the samples, the 60 mm integrating sphere accessory was used and an auto zero was performed before obtaining any results; scans were performed over wavelengths between 300 and 800 nm.

#### Description of colour

Owing to the complex, often subjective nature of colour description, where possible, colours will be described visually in broad hue terms, and using the tristimulus values x, y, Y the CIELab values L*, a*, b*, and the LCh values L, C and h* [5]. All nine values are provided to allow for maximum comparison with the literature. Owing to the complexities in the variation of L, C, h values, they are provided, but not directly compared. Colours seen in tables of data are merely indicative of hue and not exact representations of the colours achieved. Values were calculated from UV/Vis spectra using Lazurite [1], under D65 illumination, and the 1931 2° observer. To produce representative samples when using microspectroscopy, four random particles of each sample were analysed. Unless otherwise stated, values given are an average of these four sets of data.

### Materials

A UK-sourced (South Devon) highly kaolinitic ball clay was used as supplied. Mineralogical analysis *via* X-ray powder diffractometry gave a high kaolinite content (90%), with the residue as Muscovite (6%) and a low level of quartz (<2%). Chemical analysis by wavelength dispersive XRF gave the following composition: SiO_2_ (46.20%), TiO_2_ (0.61%), Al_2_O_3_ (37.72%), Fe_2_O_3_ (0.97%), CaO (0.13%), MgO (0.18%), Na_2_O (0.08%), with a small amount of carbonaceous matter (0.61%). The clay was off-white in colour owing to staining by low levels of iron oxy(hydroxide) [demonstrated by significant lightening of hue on reductive leaching with sodium dithionite/aqueous sulfuric acid] and lignitic (carbonaceous) matter. Sodium carbonate (Fisher) and sulfur (Acros) were used as purchased (without further purification). Bitumen was provided as bitumen emulsion (BS434 Class K1-40) from RMC ReadyPak, Bristol (UK).

#### Optimum Syntheses for Ultramarine Blue

Method A (Used for Sample 44). Kaolin or china clay (5.00 g) was heated overnight in a furnace at 600°C. Activated *meta*-kaolin (1.00 g) was allowed to cool to room temperature and immediately mixed with anhydrous sodium carbonate (1.00 g, 9.4 mmol.), sulphur (0.60 g, 18.7 mmol.) and bitumen emulsion (0.12 g). The resulting grey paste was ground for approximately 20 minutes with an agate pestle and mortar until a pale yellow-grey homogeneous powder was achieved. Pellets were made from 0.30 g batches of the bulk in a pelletiser and were placed within an alumina crucible and a second crucible was placed on top as a fitting lid to limit air ingress. The crucible was placed into a pre-heated furnace at 750°C and left undisturbed for 4 hours and then removed from the furnace; the lid was left in place until the crucibles had cooled to room temperature. The dark blue pellets were then removed, scraped clean and ground to yield a vivid green/blue ultramarine. CIE *xyY* 0.31215, 0.2617, 0.4257; CIE L*a*b* 3.8455, 4.2673, −3.3033; CIE *LCh* 3.8455, 5.3963, 322.256.

Method B (Used for Sample 45). Kaolin or china clay (5.00 g) was heated overnight in a furnace at 600°C. Activated *meta*-kaolin (1.00 g) was allowed to cool to room temperature and immediately mixed with anhydrous sodium carbonate (1.00 g, 9.4 mmol.), sulphur (0.60 g, 18.7 mmol.) and bitumen emulsion (0.12 g). The resulting grey paste was ground for approximately 20 minutes with an agate pestle and mortar until a pale yellow-grey homogeneous powder was achieved. Pellets were made from 0.30 g batches of the bulk in a pelletiser and were placed within an alumina crucible and a second crucible was placed on top as a fitting lid to limit air ingress. The crucible was placed into a pre-heated furnace at 750°C and left undisturbed for 4 hours. The furnace was cooled at a rate of 10 K minute^−1^ to 500°C, the top crucible was removed from the lower crucible containing the sample, and the sample left in the furnace at 500°C for a further 2 hours. After this time, the crucible and sample were removed from the furnace and allowed to cool to room temperature. The dark blue pellets were then removed, scraped clean and ground to yield a vivid blue ultramarine. CIE *xyY* 0.2945, 0.2219, 0.4836; CIE L*a*b* 4.3682, 7.6934, −7.6772; CIE *LCh* 4.3862, 10.869, 315.063.

Method C (Used for Sample 46). Kaolin or china clay (5.00 g) was heated overnight in a furnace at 600°C. Activated *meta*-kaolin (1.00 g) was allowed to cool to room temperature and immediately mixed with anhydrous sodium carbonate (1.00 g, 9.4 mmol.), sulphur (0.60 g, 18.7 mmol.) and bitumen emulsion (0.12 g). The resulting grey paste was ground for approximately 20 minutes with an agate pestle and mortar until a pale yellow-grey homogeneous powder was achieved. Pellets were made from 0.30 g batches of the bulk in a pelletiser and were placed within an alumina crucible and a second crucible was placed on top as a fitting lid to limit air ingress. The crucible was placed into a pre-heated furnace at 750°C and left undisturbed for 4 hours. The furnace was cooled at a rate of 10 K minute^−1^ to 500°C, the top crucible was removed from the lower crucible containing the sample. Sulphur (0.30 g, 0.5 mmol., 10% w/w) was added on top of the pellet (caution: ignition) and the sample left in the furnace at 500°C for a further 2 hours. After this time, the crucible and sample were removed from the furnace and allowed to cool to room temperature. The dark blue pellets were then removed, scraped clean and ground to yield a vivid blue ultramarine. CIE *xyY* 0.3098, 0.2562, 0.4340; CIE L*a*b* 3.9024, 4.6519, −3.8126; CIE *LCh* 3.9204, 6.0147, 320.662.

## Results and Discussion

**Figure 2:**
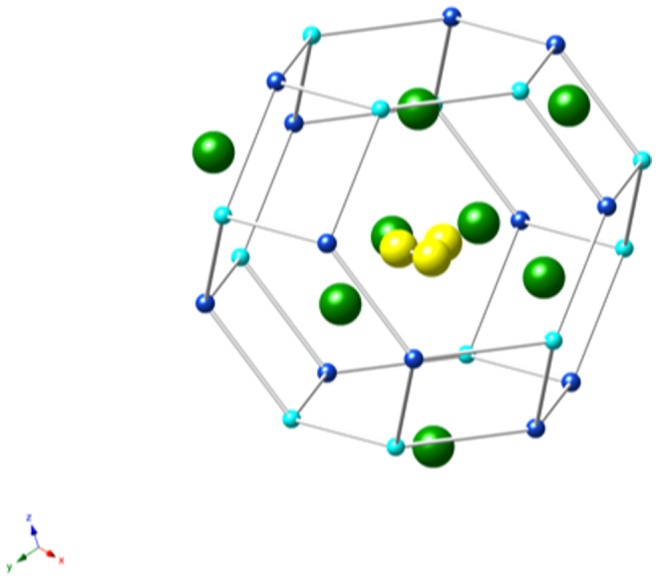
The diagram shows a single sodalite cage containing an S_3_^−^ ion. Na ions are green spheres inside the framework. The cage itself is made up of alternating Si (light blue) and Al (dark blue) tetrahedral atoms, redrawn from reference 6.

Ultramarine has the general chemical formula Na_8-x_ [SiAlO_4_]_6_.[S_2_,S_3_,SO_4_,Cl]_2−x_ and ideally the cation ratio Na_7.5_Si_6_Al_6_ [6]. The structure consists of an aluminosilicate framework, constructed from sodalite cages, containing sodium cations and sulphur-based anions (Figure 2). Industrially, various silicon compositions have been exploited to provide a range of tones, from a green-blue, to a red-blue [7]. However, the naturally occurring mineral has an aluminium: silicon ratio of around 1:1, thus silicon is not the dominant component in colour determination in the mineral [8]. Current opinion is that the colour arises from the presence of the polysulphide anions, S_2_^−^ and S_3_^−^ [1], [2], [6], [7]; the electronic structure of sulphur allows for the formation of unusual polyatomics. Although the sulphur ion, S^−^, is stable in aqueous solution, two sulphur atoms can form a co-ordinate covalent single bond. This S_2_ molecule can accept electrons and the result is the S_2_^−^ ion; sulphur can aggregate further to S_3_^−^ and even larger polysulphide anions. The S_3_^−^/S_2_^−^ ratio could also control the particular shade achieved because whilst S_3_^−^ is the key chromophore within the ultramarine blue system, absorbing in the yellow/orange region at around 590–610 nm [8], [10]–[12] imparting a blue colour on the pigment, S_2_^−^ is also usually present. The ultraviolet-visible spectrum of S_2_^−^ indicates that it could impart a yellow tone to the ultramarine as it has a significant absorption at around 380 nm [8], [11], [12]. The absorption coefficient of S_2_^−^ is much less than that of S_3_^−^ [8], so its effect does not dominate, but remains visible. S_2_^−^ is much more prevalent in ultramarine green, although not dominant. In this case the increase in S_2_^−^ concentration leads to the green tone by combination with the blue derived from a lower quantity of S_3_^−^ absorptions, thus supporting that the S_3_^−^/S_2_^−^ ratio is key to the produced colour. Additionally, the rare ultramarine yellow contains a majority contribution of S_2_^−^ [10], further strengthening theories behind the origin of the yellow tones in ultramarine green.

In the current work, a number of literatures sources were studied in an attempt to collate as much information as possible about the historical synthesis of ultramarine [7], [11], [13]–[20] and the optimum preparations described above in the experimental section were the outcome of many experiments (based on several published methods). It is largely agreed [2], [8], [13]–[17] that the main starting materials for ultramarine are kaolin (china clay) – an aluminosilicate, sulphur, sodium carbonate and a reducing agent containing carbon, often quoted as charcoal in historical (sometimes non-scientific) texts [13], [17], [19]. However, there are several varying literature recipes for the synthesis of ultramarine, and prior to commencing the preparative chemistry, an evaluation of the similarities and differences between the synthetic approaches was performed (although this was not without difficulty due to the great variation in quantities, times and temperatures quoted). In some instances [13] the methods reported are generally lacking in key details or indeed are factually incorrect and thus highly misleading. Other procedures attributed by Riffault, Vergnaud and Toussaint to Gmelin, Tremon, and Winterfield variously mention the need for a ‘well fitting cover’ or that the material should be ‘packed’ during the first calcinations – both key features for the ultimate success of the synthesis – but failed to discuss or describe the nature of the reducing species. For instance, the Weger process uses a paste (thus hinting at the need for reagents to be formed into a pellet), but does not specify that the second calcinations should be ‘expose [*sic*] to air’ and again does not discuss the reducing species.

### Preparation of ultramarine according to the procedure of Riffault, Vernaud and Toussaint

Several accounts describe differences in tones that can be achieved in ultramarine but while Riffault, Vergnaud and Toussaint reported that different compositions of the starting materials yielded different tones in the product, denoted as tone I, II, and III (Table 1), the identity of the tones and differences between them was omitted (*i.e.* from the text, it cannot be deduced whether I is the darkest or lightest shade of ultramarine obtained). The correct temperature for calcinations in the furnace is described as when the furnace is a ‘light red, or incipient white heat’, and that extra sulphur should be added when the furnace has cooled ‘to the point at which a small quantity of sulphur, projected through the upper opening, will become inflamed’ [13]. Whilst the correct temperature ranges may be inferred, this is not particularly reproducible.

**Table 1:** Proportions (expressed as ‘parts’) taken from *The Gentele Process* for Ultramarine Blue Synthesis [13].

	Kaolin	Sodium Carbonate	Sodium Sulphate	Coal	Sulphur
I	100	-	83/100	17	-
II	100	100	-	12	60
III	100	41	41	17	13

Consequently, the three different proportions described by Riffault *et al.* were all made-up and ground in a pestle and mortar to achieve homogeneity of the mixture, with amorphous carbon used as the reducing agent. It should be note that the text of ‘*A Practical Treatise on the Manufacture of Colors for Painting’* is often verbose and often difficult to understand. For example, the description of the different syntheses of ultramarine spans sixty-five pages and key details are often difficult to pin-point amongst lengthy descriptions of the oven's dimensions and the layout of crucibles inside the furnace. Little attempt is made to critique or compare the twelve different preparative methods it contains. The original source suggests that heating at ‘the proper degree of high temperature, without the contact of air’ should be maintained between seven and ten hours and then the sample cooled slightly to a temperature sufficient to ignite additional sulphur added to the sample (now deemed to be around 500°C). The reducing atmosphere was initially assumed to have probably been merely a sealed vessel or the use of a vessel with restricted air-flow as colour chemists and alchemists at the time would not have had access to gaseous, oxygen-free reducing atmospheres, such as a nitrogen flow. This was supported by diagrams (Reference 13, page 313) showing a closed crucible as the heating vessel. The ‘cherry-red’ temperature described in the text was estimated to be between 700 and 900°C, although this could vary depending on the material used to make the furnace. Using these temperatures, only a pale grey or pale pink was achieved. Various other times and temperatures were investigated at this stage and, in accordance with ‘*Industrial Inorganic Pigments’* [14], the kaolin was also heated prior to synthesis and the temperature monitored closely, varying times to try and obtain a blue tone in the product. Unfortunately, tones of cream, grey and pink were again formed, entirely unusable as an ultramarine pigment. Although the proportions were altered slightly, temperatures were manipulated and even a nitrogen furnace employed to achieve the reducing atmosphere, no blue-toned pigment was produced.

### Preparation of ultramarine according to the procedure of Buxbaum

Buxbaum's account [14] first suggested temperatures and the compositional changes that occur during the heating of the starting materials, thus providing a ‘scientific’ and, more importantly, a potentially reproducible synthetic scheme to follow in our work. Buxbaum discusses the varying tones of ultramarine blue that can be obtained, and provided different quantities to achieve these variations. Our preparations used proportions reported for the ‘green tone’ reported by Buxbaum (Table 2) as they mirrored more closely the other syntheses that had already been investigated in our work.

**Table 2:** Proportions (expressed as ‘parts’) taken from *Industrial Inorganic Pigments* for Ultramarine Blue Synthesis [14].

Kaolin	Sodium carbonate	Reducing species	Sulphur
32	29	4.5	34.5

Through several attempts at slightly varying preparations it was possible to produce a pigment that was blue, although this could not be described as ‘ultramarine’ and was not directly comparable to a sample of *lapis lazuli,* the tone being much more ‘green-blue’, perhaps Buxbaum’s ‘green tone’. In accordance with ‘*Industrial Inorganic Pigments’*, the kaolin was also heated prior to synthesis and the temperature monitored closely, varying times to try and obtain a blue tone in the product (Table 3). Unfortunately, tones of cream, grey and pink were again formed, entirely unusable as an ultramarine pigment. While Buxbaum highlights a number of different reducing agents that are suitable and even goes into depth about structural processes that are occurring during the calcinations, much like the more historical texts, the modern text quotes no times for the calcinations and, crucially, the text describes that the mixture is ground before calcination, and does not discuss any forms of compaction that were subsequently found to be vital to the success of the synthesis.

**Table 3:** Changes seen after varying preparative methods: Buxbaum ‘Green-tone’ recipe.

Sample	Recipe	Description	Appearance	CIE Values								
				x	y	Y	L*	a*	b*	L	C	h
36	Buxbaum Pellet	Heat at 750°C for 5 hours in air	Speckled Blue Grey	0.3476	0.3276	0.3247	2.9334	1.4907	0.4099	2.9334	1.5460	15.3737
37	Buxbaum	Heat at 750°C for 5 hours with limited air ingress	Green Blue	0.3285	0.2867	0.3848	3.4756	3.0909	-1.4113	3.4756	3.3979	335.4582
	Pellet											
38	Buxbaum Pellet	1.Heat at 750°C for 240 minutes with limited air ingress	Green Blue, little difference to above	0.3261	0.2845	0.3894	3.5174	3.2221	-1.7183	3.5174	3.6516	331.9299
		2. Cool to 500°C and heat for 2 hours										
39	Buxbaum Pellet	1.Heat at 750°C for 240 minutes with limited air ingress	Green Blue, slightly more ‘blue’ than 37 and 38	0.3218	0.2696	0.4086	3.6908	4.6485	-3.2514	3.6908	5.6727	325.0294
		2. Cool to 500°C and at 10% by weight sulphur										
		3. Maintain heat for 2 hours										
40	Buxbaum Powder	1.Heat at 750°C for 240 minutes with limited air ingress	Pale Pink	0.3581	0.3547	0.2871	2.5935	0.6969	1.1423	2.5935	1.3381	58.6139
		2. Cool to 500°C and at 10% by weight sulphur										
		3. Maintain heat for 2 hours										

### Preparation of ultramarine according to the procedures of Zerr and Rubencamp

Zerr and Rubencamp described the compositions (with quantities again described in parts, Table 4) used to synthesise ultramarine as producing light, medium and dark ultramarine [17], which gave an indication as to what to expect from the synthesis. In the preparation of ‘dark ultramarine’ a new starting material, silica, is introduced in this version of the synthesis. Furthermore, although the ratios are similar and starting materials largely familiar, according to this text the exact proportions required to produce the ‘medium’ tone are exactly the same as those quoted [13] for ultramarine tone II. It is noteworthy that when elemental sulphur is used in smaller proportions, sodium sulphate is used to make up some of the shortfall. Conversely, when sodium sulphate is used, less sodium carbonate is needed to provide the sodium to stabilise the polysulphide ions entrapped inside the lattice. Once again, the kaolin was activated by heating at 650°C overnight before use.

**Table 4:** Proportions (expressed as ‘parts’) taken from *A Treatise on Colour Manufacture* for Ultramarine Blue Synthesis [17].

	Kaolin	Sodium Carbonate	Sodium Sulphate	Carbon	Sulphur	Silica
Light	100	9	12	25	16	-
Medium	100	100	-	12	60	-
Dark	100	103	-	4	117	16

Mixtures were made up according to the Zerr and Rubencamp recipe for the medium tone of ultramarine, homogenized by hand, with an agate pestle and mortar and made into circular pellets using a Specac pelletizer. The pellets create a ‘bound’ sample, minimising the sulphur that can sublime from the mixture, and creating a highly reducing atmosphere at the centre of the pellet. Zerr and Rubencamp describe the calcination process as heating to a ‘red-white’ heat for up to nine hours, depending on the proportion of sodium carbonate, and then cooling the sample slightly to temperatures that will ignite sulphur (now known to be around 500°C). At this point the text describes the addition of around 10% weight sulphur to the sample and further heating for 30-minute increments with visual checks. Although a total time is not quoted, nor a specific method of addition, the literature suggests 45 minutes of further heating after the sulphur has all burned off. The description adds the cautionary note that heating should be stopped if white specks appear in the sample [17], supported by a quote from Riffault *et al.*, ‘ the pigment will lose its qualities if the treatment be longer continued’ [13]. Zerr and Rubencamp describe several coal sources, including pitch and ‘colophony’ that is derived from tree resin, both ‘sticky’ reducing agents that would hold the mixture together. There is a fleeting mention of the ‘pressed down’ mixture and the ‘exclusion of air’ but the main body of text describing the calcinations ends with ‘no definite instructions can be given on this point’ referring to the times of calcination.

The ‘red heat’ that has already been mentioned is the only description of the adequate temperature for calcination. Even if one assumes that the ‘red-white’ heat, rather than the ‘cherry-red’, described by Riffault *et al.*, is the same as the 750°C used by Weller *et al.* [7], then the times are quite different for the first calcination (*e.g.* half those used in other publications). The pellets formed from the Zerr and Rubencamp ‘Medium’ recipe were heated for seven hours, with a lid on the crucible, as per the text. The lids were then removed, the furnace was allowed to cool to 500°C, and around 10% sulphur (by weight) added; heating at this temperature was continued for 30 minutes. The resulting pellet was grey but when broken into pieces with a pestle and mortar, had a bright blue centre, approaching an ultramarine-like blue. This suggested that the proportions used were approximately correct but that the conditions required some optimisation.

Whilst times and temperatures are scarce amongst the literature, Zerr and Rubencamp are specific about the addition of the sulphur, stating that the mixture and sulphur are placed in the furnace and subjected to heat, and that sulphuric acid forms that oxidises the green ultramarine. The description goes on to state usefully that the mixture should be ‘*stirred every half an hour and observed…*’ and ‘*…maintain heat for 45 minutes after sulphur has burned off*’ and then left to cool over two and a half hours. However, along with these very useful details (to assist the reproduction of the synthesis), less specific areas of text, indicate that *‘…to ascertain the precise moment for adding the first portion of sulphur a lump of sulphur must be thrown on the charge when the latter is heated right through; if the sulphur ignites at once the first portion may be added’.*

In an attempt to pin-point the correct time and conditions of both calcinations to give a viable ultramarine tone, the three Zerr and Rubencamp recipes, light, medium and dark, were synthesized on a *ca.* 4 g scale and multiple pellets were produced. The first calcination was performed at 750°C as this value was common to the historical texts could agree on, and had produced a blue-toned product from earlier tests. Multiple test reactions were carried out varying the primary, reducing calcination time and the secondary, oxidizing calcination time and conditions. The test syntheses cover variations seen across preparations described in *‘A Practical Treatise on the Manufacture of Colors for Painting’*, *‘The Pigment Handbook’*, *‘A Treatise on Colour Manufacture’* and the modern article by Weller *et al.* – the results are recorded below.

### The Zerr and Rubencamp ‘Light Recipe’ and ‘Dark Recipe’

For the ‘Light Recipe’ – the observed colour and the resulting UV-Visible spectra showed little variation and this is mirrored by the corresponding CIE values (Table 5). These values show that the light recipe shows little progression in the colour of the pigment throughout the heating process, which is attributed to the fact that there is very little sulphur in the recipe (and what is present merely sublimes in the high temperatures of the furnace).

**Table 5:** Changes observed after each calcination for the Zerr and Rubencamp ‘light recipe’ time course.

Sample	Description	Appearance	1931 CIE Values								
			x	y	Y	L*	a*	b*	L	C	h
1	Heat at 750°C for 30 minutes with limited air ingress	Medium Grey Pellet	0.3506	0.3483	0.3011	2.7201	0.6964	0.9629	2.7201	1.1883	54.1230
2	Heat at 750°C for 60 minutes with limited air ingress	Very Pale Grey	0.3664	0.3622	0.2715	2.4521	0.6801	1.3068	2.4521	1.4732	62.5083
3	Heat at 750°C for 90 minutes with limited air ingress	Very Pale Grey	0.3634	0.3585	0.2781	2.5125	0.7042	1.1494	2.5125	1.3480	58.5073
4	Heat at 750°C for 180 minutes with limited air ingress	Very Pale Grey	0.3582	0.3539	0.2879	2.6005	0.7250	1.1146	2.6005	1.3296	56.9567
5	Heat at 750°C for 240 minutes with limited air ingress	Very Pale Grey	0.3639	0.3596	0.2765	2.4974	0.6984	1.2323	2.4974	1.4165	60.4577
6	Heat at 750°C for 300 minutes with air ingress	Very Pale Grey	0.3688	0.3629	0.2683	2.4232	0.7238	1.3182	2.4232	1.5039	61.2279
7	Heat at 750°C for 30minutes with air ingress	Very Pale Grey	0.3423	0.3407	0.3170	2.8638	0.7047	0.7092	2.8638	0.9998	45.1835
8	Heat at 750°C for 60 minutes with air ingress	Very Pale Grey	0.3561	0.3548	0.2892	2.6121	0.6313	1.1034	2.6121	1.2712	60.2220
9	Sample prior to heating	Beige/Grey Powder	0.3689	0.3626	0.2685	2.4254	0.7485	1.2292	2.4254	1.4391	58.6612

In common with the light recipe, the ‘Dark Recipe’ also showed little success in producing a blue pigment (Table 6), which supports the findings of previous work, wherein it is stated that the colour strength and purity is largely controlled by the amount of sodium sites that can accommodate the sulphur [11]. Therefore, whilst the dark recipe contains much more sulphur, it does not contain a proportional increase in sodium sites that could balance the polysulphides. The chromaticity diagram produced from the spectral data revealed that the newly-produced pigments showed little change from the original, un-calcined mixture. The washing step employed in other literature sources [7], [13], [18] did not appear to have any beneficial effects on the pigment so it was not used for the other samples and discontinued for other experiments.

**Table 6:** Changes observed after each calcination for the Zerr and Rubencamp‘dark recipe’ time course.

Sample	Description	Colour	CIE Values								
			x	y	Y	L*	a*	b*	L	c	h
27	Heat at 750°C for 30 minutes with limited air ingress	Grey	0.3734	0.3656	0.2610	2.3575	0.7415	1.3844	2.3575	1.5704	61.8252
28	Heat at 750°C for 60 minutes with limited air ingress	Grey	0.3685	0.3583	0.2731	2.4670	0.8752	1.2700	2.4670	1.5423	55.4285
29	Heat at 750°C for 90 minutes with limited air ingress	Grey	0.3771	0.3660	0.2569	2.3204	0.8420	1.4155	2.3204	1.6470	59.2557
30	Heat at 750°C for 180 minutes with limited air ingress	Pale Grey	0.3703	0.3599	0.2698	2.4373	0.8600	1.2922	2.4373	1.5522	56.3550
31	Heat at 750°C for 240 minutes with limited air ingress	Beige/Grey Powder	0.3792	0.3678	0.2530	2.2853	0.8352	1.4497	2.2853	1.6731	60.0546
32	Heat at 750°C for 300 minutes with air ingress	Brown	0.3682	0.3634	0.2684	2.4249	0.6938	1.3394	2.4249	1.5084	62.6144
33	Heat at 750°C for 30 minutes with air ingress	Pale Grey	0.3640	0.3556	0.2805	2.5335	0.8389	1.1802	2.5335	1.4480	54.5960
34	Heat at 750°C for 60 minutes with air ingress	Pale Grey	0.3640	0.3572	0.2788	2.5184	0.7838	1.2234	2.5184	1.4530	57.3549
35	Sample prior to heating	Yellow/Grey	0.3730	0.3694	0.2576	2.3267	0.6199	1.3804	2.3267	1.5132	65.8179

### The Zerr and Rubencamp ‘Medium Recipe’

In contrast to the previous preparative methods, during the ‘Medium Recipe’ the pigment colour developed during the time course with continued heating (Table 7), with the observed colour brightening at four hours during the first calcination and beginning to dull after this point. Further heating at this temperature, as suggested by Weller *et al.* [7], Zerr and Rubencamp [17] and Moser [19], both in the presence and absence of air, and in both pelletised and powdered forms, provided a product but one that was not useful as a blue pigment. From which it was deduced that the duration of the calcinations has a large effect on the observed colour of the pigment and thus its viability as a useful tone.

**Table 7:** Changes observed after each calcination for the Zerr and Rubencamp ‘medium recipe’ time course.

Sample	Recipe	Description	Appearance	CIE Values								
				x	y	Y	L*	a*	b*	L	C	h
10	Medium	Heat at 750°C for 30 minutes with limited air ingress	Medium Grey	0.3516	0.3401	0.3083	2.7846	1.0530	0.7901	2.7846	1.3165	36.8821
11	Medium	Heat at 750°C for 60 minutes with limited air ingress	Medium Grey	0.3421	0.3183	0.3396	3.0678	1.7937	0.0341	3.0678	1.7940	1.0894
12	Medium	Heat at 750°C for 90 minutes with limited air ingress	Medium Grey	0.3436	0.3256	0.3308	2.9883	1.4274	0.3395	2.9883	1.4672	13.3800
13	Medium	Heat at 750°C for 180 minutes with limited air ingress	Grey Blue	0.3336	0.2987	0.3677	3.3213	2.5696	−0.9325	3.3213	2.7335	340.054
**14**	**Medium**	**Heat at 750°C for** **240** **minutes with** **limited air ingress**	**Vivid** **ultramarine**	**0.3212**	**0.2741**	**0.4046**	**3.6551**	**3.7398**	**−2.3991**	**3.6551**	**4.4432**	**327.319**
**15**	**Medium**	**Heat at 750°C for** **300** **minutes with** **limited air ingress**	**Dull** **ultramarine**	**0.3054**	**0.2544**	**0.4402**	**3.9765**	**4.5278**	**−4.0599**	**3.9765**	**6.0814**	**318.119**
16	Medium	Heat at 750°C for 960 minutes with limited air ingress	Off-white	0.3623	0.3575	0.2802	2.5313	0.7256	1.2144	2.5313	1.4146	59.1435
17	Medium	Heat at 750°C for 3600 minutes with limited air ingress	Peach	0.3604	0.3567	0.2829	2.5553	0.6968	1.1908	2.5553	1.3797	59.6663
18	Medium as powder	Heat at 750°C for 3600 minutes with limited air ingress	Off-white	0.3586	0.3561	0.2853	2.5769	0.6573	1.1570	2.5769	1.3307	60.3988
19	Medium	Heat at 750°C for 30 minutes with air ingress	Grey	0.3447	0.3245	0.3307	2.9876	1.5372	0.3008	2.9876	1.5663	11.0730
20	Medium	Heat at 750°C for 60 minutes with air ingress	Inner Pellet, Blue/Grey	0.3450	0.3190	0.3360	3.0349	1.8304	0.1175	3.0349	1.8342	3.6734
21	Medium	As above	Outer Pellet, Speckled Orange and White	0.3631	0.3559	0.2810	2.5386	0.7818	1.1466	2.5386	1.3877	55.7129
**22**	**Medium**	**Heat at 750°C for** **240** **minutes with** **air ingress**	**Inner Pellet, Vivid** **ultramarine Blue**	**0.3325**	**0.3035**	**0.3640**	**3.2884**	**2.3706**	**−0.8594**	**3.2884**	**2.5215**	**340.073**
23	Medium	As above	Outer Pellet, Speckled Peach and White	0.3526	0.3492	0.2981	2.6931	0.7201	0.9864	2.6931	1.2212	53.8690
**24**	**Medium**	**1.Heat at 750°C for 240** **minutes with limited air ingress**	**ultramarineSlightly Duller than Sample 22**	**0.3156**	**0.2701**	**0.4142**	**3.7418**	**3.7429**	**−2.6880**	**3.7418**	**4.6082**	**324.315**
		2. Cool to 500°C and heat for 2 hours										
**25**	**Medium**	**1.Heat at 750°C for 240** **minutes with limited air ingress**	**ultramarine** **Very Similar to Sample 22**	**0.3016**	**0.2359**	**0.4625**	**4.1774**	**6.3101**	**−5.9655**	**4.1774**	**8.6835**	**316.607**
		2. Cool to 500°C and at 10% by weight sulphur										
		3. Maintain heat for 2 hours										
26	Medium	Raw Mix	Pale Yellow	0.3584	0.3550	0.2865	2.5883	0.6879	1.1181	2.5883	1.3127	0.3255

**Figure 3:**
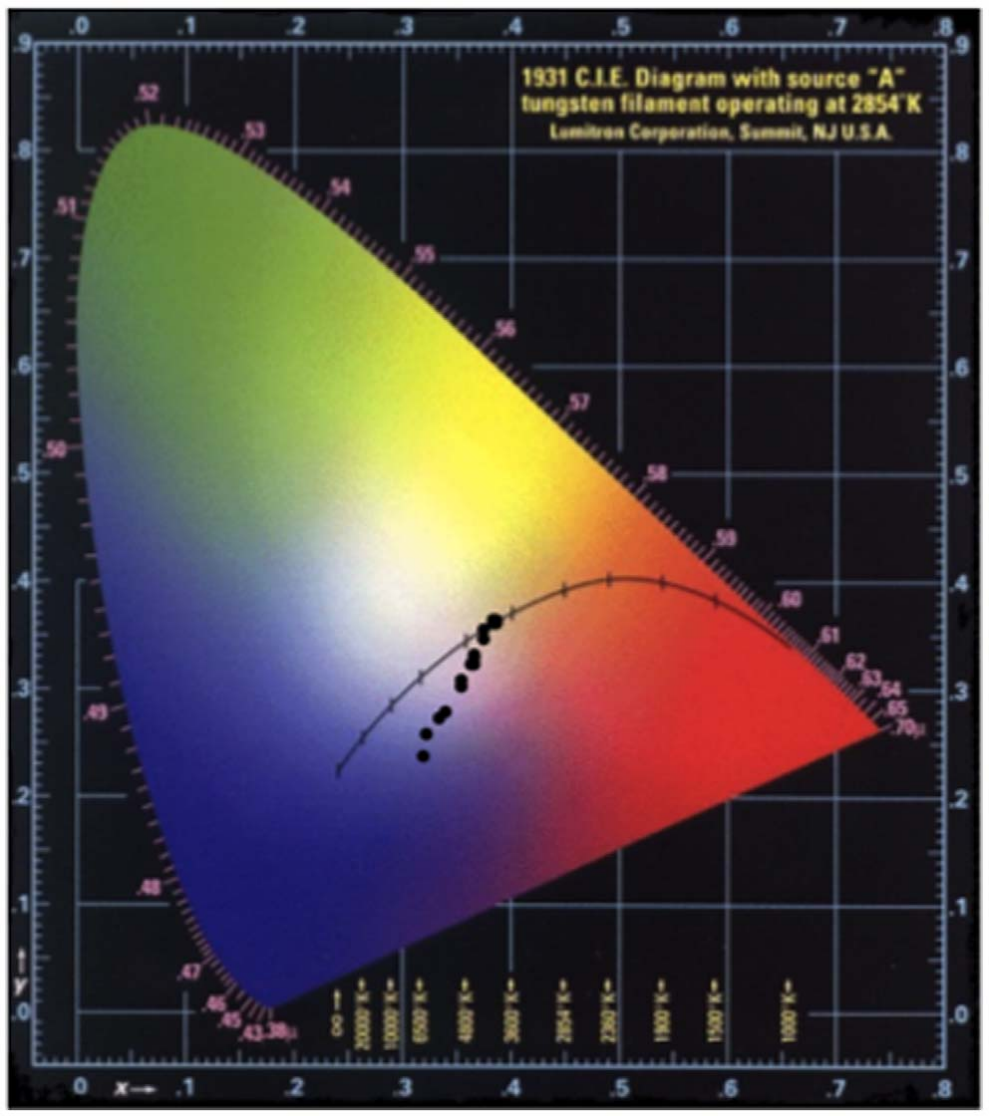
Chromaticity diagram plotting x,y values of products from the Zerr and Rubencamp ‘medium recipe’ reduced synthesis.

**Figure 4:**
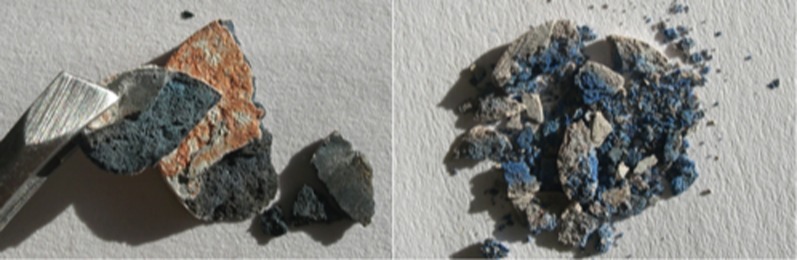
Broken pellets of (a) samples 20 and 21 and (b) samples 22 and 23.

The pellets that showed the most promising blue tone were compared to a small piece of *lapis lazuli*. In fact, when the pellets first emerge from the furnace and are allowed to cool, the surface appears mottled and ‘veined’, much like the naturally occurring mineral. Significantly, in comparison to the chromaticity diagram for the products of the light recipe, the pigments are now approaching the ‘blue’ region of the xy colour space (Figure 3). Of particular interest here, are the differences in the colours seen in the preparation that allowed air ingress. The inner surfaces of the pellets are a completely different colour to that seen on the surface of the pellet (Figure 4a and 4b); in the former the surface of the pellet remains a mottled orange and pale peach.

**Figure 5:**
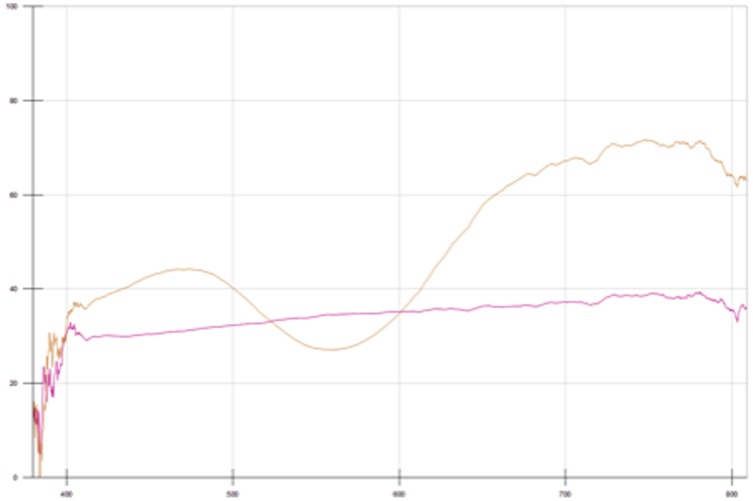
UV-Vis spectra for sample 22 pellet interior (orange) and sample 23 pellet exterior (pink) after 4 hours.

As the initial development of the colour will not tolerate air, it is postulated that the inside of the pellet was subject to an oxygen deficient atmosphere, from which sulphur could neither sublime nor be oxidised. Consequently, the inner sample would undergo similar reductive processes to those occurring in samples 10 to 15, whilst the outside of the pellet loses what sulphur it contains to sublimation and over-oxidation at high temperatures. The resultant spectra show that whilst the outsides of the pellets remain largely useless as blue pigments, the inside of the mixture begins to form a usable tone at just 1 hour of calcination and is comparable to the colours obtained from the reductive conditions at four hours (Figure 5).

This is a significant point for two particular reasons; firstly, the formation of at least some usable pigment at the centre of the pellet may go some way to explaining why reducing conditions are not quoted in all preparations. For industrial purposes large ‘bricks’ of pigment mix were, and still are, used. Therefore, allowing for removal of the useless outer surface of the brick, the core would still be useful as a ‘reservoir’ of ultramarine pigment. Secondly, the formation of the blue tone inside the pigment suggests that the ‘light’ recipe was not merely subject to incorrect conditions, but that there is indeed something fundamentally wrong with the proportions, as we know that at high temperatures, using pellets, ultramarine formation can still be propagated, even in oxidising conditions.

Samples 14, 24 and 25 were used to investigate a variation in the synthesis that cropped up in the literature [13], [17], wherein additional powdered sulphur is added to the sample after the first calcination, once the sample and furnace have been allowed to cool to a temperature sufficient to ignite the sulphur, around 500°C. The intention was to run sample 14 with just the first calcination with the optimised conditions of 750°C for 4 hours with limited air ingress. Sample 24 was subject to this first calcination but then cooled at around 10 K per minute until the temperature reached 500°C. The sample was then held at this temperature for two hours. Sample 25 was heated for the optimum four hours at 750 C and then cooled at the previous rate to 500°C. However, at this point around 10% by weight excess sulphur was added on top of the pellet and heating at 500°C was then continued for two hours.

**Figure 6:**
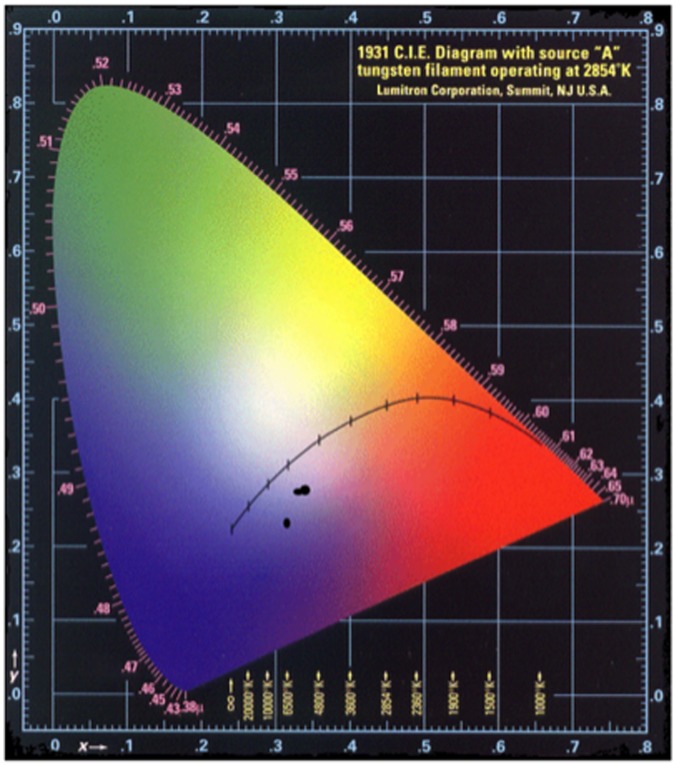
Chromaticity diagram showing viable blue pigment samples 14, 24, and 25.

Visually, the pigments looked very similar, but plotting their spectral characteristics on the chromaticity diagram shows that sample 25 is substantially closer to the blue region of the diagram (Figure 6) than the others. When analysing the data presented it is important to provide a representative data set. Therefore, when taking samples, a range of particle types was analysed, which leads to data that can be more accurately discussed. However, in this case, the use of ‘average data’ has highlighted an interesting point. When viewed with the naked eye, the samples all look quite similar, but, when seen under a microscope the samples differ: there is a greater distribution of particle types in the earlier two samples and this has had an effect on the averaged results. Whilst individual particles in samples 14 and 24 were indeed ultramarine blue, falling more towards the blue region of the diagram, by using the average value we see a more representative view of the sample as a whole. Effectively, un-reacted grey particles reduce the optimum blue region in xy chromaticity diagram.

As the prolonged heating exposes the sample to further oxidation it is hypothesised that the pellet is exposed to more uniform conditions, inside and outside the pellet in sample 24. When sulphur is added to the 500°C sample, it immediately ignites, possibly aiding the sodium carbonate transition, and providing more sites to balance the polysulphide anions, whilst favouring the SO_2_ formation. This, in turn, forms more polysulphides for trapping in the lattice, converting any previously un-reacted material into ultramarine, effectively providing more uniformity in the sample. Once ground, the pellets form fine powders that could indeed be used as a pigment, but as previously noted [13] the colour achieved is somewhat diminished by grinding; the larger the particles the brighter the pigments appear. This could also be attributable to the degree of homogeneity in the sample – increased grinding produces a more even tone, but it also incorporates the un-reacted portions of the sample, as well as some of the ‘charred’ surface of the pellet, making the colour duller. The best approximation to the piece of the natural mineral was therefore formed from samples 14, 24 and 25 by eye. Hence, the ‘Medium’ recipe can be assumed to be a good record of the correct proportions of starting materials to produce ultramarine of a desirable tone.

### Investigation of the Effect of Sulphur Content on the Colorimetric Properties of Ultramarine

It is well known that the intense blue seen in ultramarine is largely due to the trapped polysulphide ions, specifically S_3_^−^, but having achieved a blue tone in the product using the Zerr and Rubencamp ‘Medium recipe’ a short study was undertaken to determine whether simply increasing the amount of sulphur in the initial preparation would yield a more intense blue tone in the pigment. While maintaining the ratio of other components in the mixture in line with the procedure reported by Zerr and Rubencamp [17], the proportion of sulphur in the mixture was increased incrementally to a final value that was double the initial level and at each stage the pigment was monitored closely. Recipes were assigned a number to minimise confusion (Table 8) *N.B.*, the Zerr and Rubencamp medium recipe is method 1.

**Table 8:** Proportions of reactants used in recipes to examine the effect of sulphur content.

Recipe	Kaolin(parts)	Sodium carbonate (parts)	Bitumen emulsion (parts)	Sulphur (parts)	4 pellets (g)
1	100	100	12	60	1.36
2	100	100	12	80	1.38
3	100	100	12	100	1.40
4	100	100	12	120	1.42

*N.B.*, Recipe 1 is the ‘Zerr and Rubencamp Medium Recipe’.

This short study would further test the hypothesis that the colour intensity of the pigment is proportional not only to the sulphur content, but also to the number of sodium sites that can accommodate the polysulphide anions. In order to monitor important colour changes, the pigment was removed at four different junctures:

- after the mixture had been exposed to 750°C for four hours with air being allowed to reach the sample;
- after the mixture had been exposed to 750°C for four hours with limited air ingress, by means of a tight fitting but not sealed lid;
- after the pellet had been exposed to 750°C for four hours, but then allowed to cool at ca. 10 K minute^−1^ to 500°C and maintained at this final temperature for 2 hours;
- after the pellet had been exposed to 750°C for 4 hours, but then allowed to cool at *ca.* 10 K minute^−1^ to 500°C, additional sulphur (10% w/w) was placed on top of the pellet and the mixture maintained at this final temperature for 2 hours.

Points (a) and (b) were chosen to investigate how the reducing atmosphere alters the colour of the produced pigment. The third time point was chosen to determine whether the second calcination had an effect on the colour of the pigment. Although largely included in synthetic schemes, this step is omitted from the Moser description [19]. The fourth and final time-point was used to investigate a variation in the synthesis wherein there is a further addition of around 10% sulphur added to the cooled sample. All of the samples were removed from the furnace and allowed to cool to room temperature. The colours of the products arising from the different recipes are given in Table 9.

**Table 9:** Changes Seen After Each Calcination for different recipes.

Sample	Recipe	Description	Appearance	CIE Values								
				x	y	Y	L*	a*	b*	L	c	h
43	1	1	Peach and Grey	0.3501	0.3363	0.3136	2.8330	1.1911	0.6552	2.8330	1.3594	28.8138
44	1	2	Green Blue	0.3125	0.2617	0.4257	3.8455	4.2673	**−**3.3033	3.8455	5.3964	322.256
**45**	**1**	**3**	**Vivid Ultramarine**	**0.2945**	**0.2219**	**0.4836**	**4.3682**	**7.6943**	**−7.6772**	**4.3682**	**10.869**	**315.063**
**46**	**1**	**4**	**Vivid Ultramarine**	**0.3098**	**0.2562**	**0.4340**	**3.9204**	**4.6519**	**−3.8126**	**3.9204**	**6.0147**	**320.662**
47	2	1	Speckled Blue Grey	0.3671	0.3571	0.2759	2.4919	0.8784	1.2367	2.4919	1.5169	54.6152
48	2	2	Pale Grey Blue	0.3493	0.3199	0.3307	2.9876	2.0271	0.0599	2.9876	2.0280	1.6921
49	2	3	Pale Grey Blue	0.3564	0.3332	0.3105	2.8043	1.5436	0.6445	2.8043	1.6727	22.6609
**50**	**2**	**4**	**Vivid Ultramarine**	**0.3150**	**0.2639**	**0.4211**	**3.8034**	**4.6616**	**−3.8392**	**3.8034**	**6.0390**	**320.525**
51	3	1	Pale Grey	0.3565	0.3507	0.2929	2.6455	0.7960	1.0563	2.6455	1.3226	53.0009
52	3	2	Dark Blue Grey	0.3483	0.3124	0.3394	3.0654	2.3101	**−**0.0348	3.0654	2.3103	359.137
53	3	3	Medium Grey Blue	0.3637	0.3523	0.2840	2.5654	0.9537	1.1380	2.5654	1.4848	50.0349
54	3	4	Speckled Grey/Ultramarine	0.3128	0.2582	0.4290	3.8753	4.6182	**−**3.5491	3.8753	5.8244	322.457
55	4	1	Peach	0.3637	0.3600	0.2763	2.4962	0.6806	1.2652	2.4962	1.4366	61.7221
56	4	2	Pale Blue Grey	0.3543	0.3421	0.3035	2.7419	1.0652	0.8716	2.7419	1.3764	39.2936
57	4	3	Pale Blue Grey	0.3479	0.3251	0.3271	2.9543	1.7250	0.1566	2.9543	1.7321	5.1886
58	4	4	Pale Blue Grey	0.3313	0.2863	0.3824	3.4539	3.3168	**−**1.4445	3.4539	3.6177	336.466

**Figure 7:**
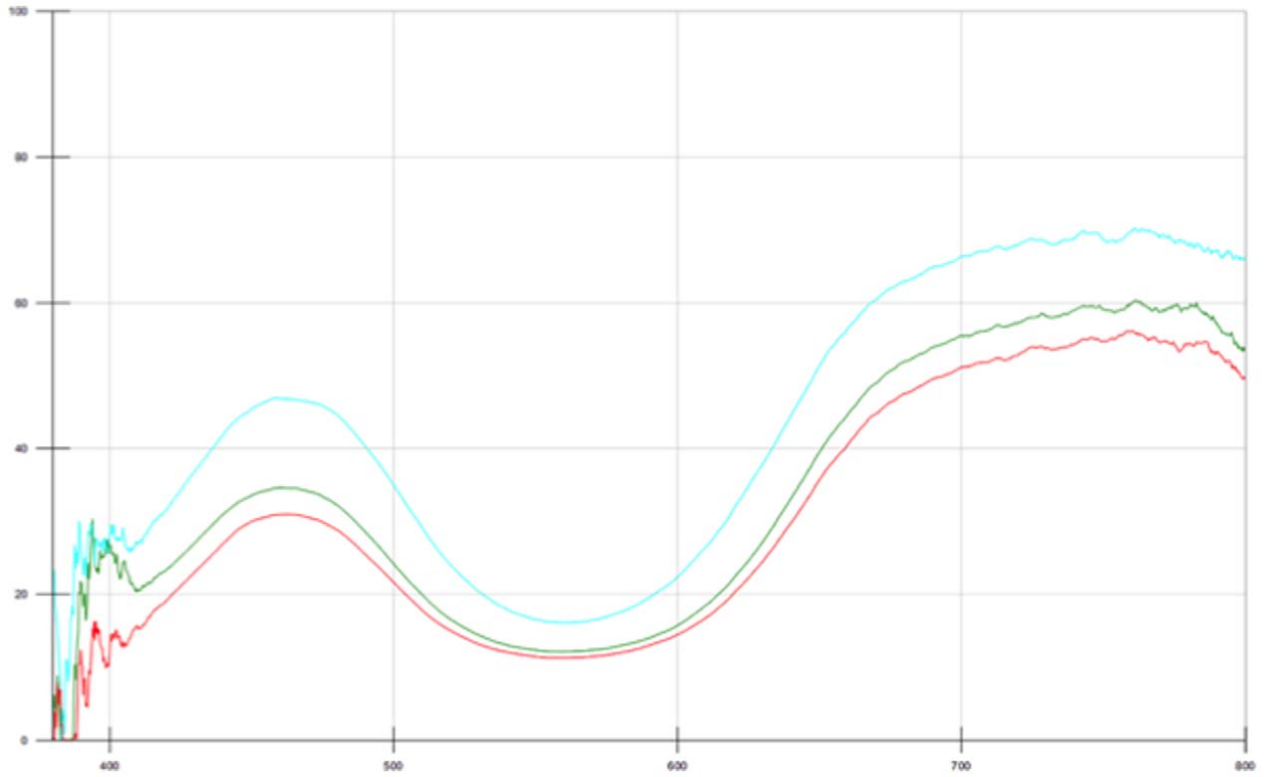
UV-Vis spectra for the viable blue pigment sample 44 as a function of time (red  = 2, green  = 3, blue  = 4).

**Figure 8:**
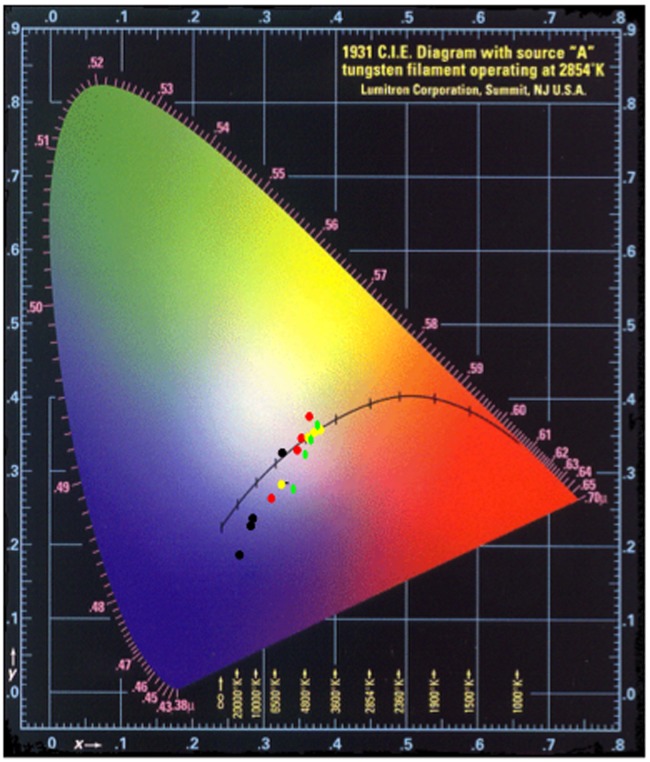
Chromaticity diagram showing development in colour with respect to time for different recipes: (• black circle) xy plot of Recipe 1 products, (• red circle) xy plot of Recipe 2 products, (• yellow circle) xy plot of Recipe 3 products, (• green circle) xy plot of Recipe 4 products.

After cooling, the surfaces of the pellets were mottled in colour and the pellet was broken up to investigate differences between the colour produced inside the pellet and at the surface. For spectral measurements, samples from the centre of the pellet were used as these would represent the majority of the usable pigment from industrial fire ‘bricks’ used historically to synthesise the pigment in bulk. This also removes much of the problem with the ‘dulling’ of the colour seen in grinding the medium recipe from previous experiments. The spectra from recipe 1 (the Zerr and Rubencamp medium recipe) show that the blue tone develops, again with increasing uniformity as the preparation increases in time. The spectra from sample 44 (Figure 7) are very similar to those obtained from the Buxbaum synthesis after the first calcination and appears to be the ‘green ultramarine’, quoted in many historical sources [13], [11], [17], [18]. As the charred outer pellet was removed, the samples were much brighter and gave a much more discernible blue pigmentation (Figure 8). However, as the sulphur content of the mixture was increased, the product became less useful as a blue pigment.

When heated in the absence of air, most of the products achieved a blue tone but it only recipe 2 (time-point 4) that approached the vivid ultramarine tones of the recipe 1 syntheses (the data are presented on a chromaticity diagram for ease of comparison, Figure 8). From the CIE xyY chromaticity diagram and observations it can be interpreted that the best proportions are, in fact, those from the text [17] [17]. These consistently produced a blue-toned, usable pigment, from the last three time-points, implying that when the correct proportions are used, changes in the calcinations are tolerated. However, the only other usable ultramarine pigment from observation was obtained using recipe 2 (time-point 4), which produced a vivid blue tone after addition of the sulphur. This would indicate that as the proportions tend away from the optimal, a successful synthesis is possible, but is more dependent on specific synthetic conditions.

This last step also suggests that excess sulphur does indeed lead to the incorporation of S_3_^-^ anions after the sodalite has been formed. The pellets that contained an increasing amount of sulphur began to take on an increasingly ‘charred’ appearance. Perhaps the sheer quantity of sulphur caused increased heat and ignition inside the pellet mix, burning the pigment rather than creating sufficient anion amounts for a usable ultramarine pigment; any extra polysulphide anions that would form would also have insufficient quantities of sodium counter ions. Therefore if increasing the sodium content in order to obtain a usable pigment, it is reasonable to suggest that more sodium should be added in the form of sodium carbonate to aid this. Whilst Weller *et al.* have investigated increasing cation content to modify the colours seen, it does not appear that sulphur was increased alongside the cation content to fill the new cation sites in the sodalite cage.

Visually, there appears very little difference in the hues seen of the different time-points from recipe 1. Nevertheless, time-point 2 appears to be ‘greener’ in tone than the two later time-points, possibly due to an increased proportion of S_2_^2−^ anions in time-point 2, which was not heated for a second time. CIE xyY data describe samples 45 and 46 as being located further into the ‘blue’ region of the chromaticity diagram, compared to sample 44, and more specifically, 45 is considerably further into the blue region (at 0.2945, 0.2219 further supporting the visual observations. The CIE L*ab data most strongly support the observation that sample 45 produces the best tone. As discussed previously, the more negative the a* value, the greener the sample, and the more negative the b* sample, the bluer it is. Sample 45 has an a* value at least 3.0 units higher than sample 44 and 46, and a b* value of at least 3.8 units lower. Therefore this describes sample 45 as the least green and most blue.

## Conclusions

To synthesise a historically accurate sample of ultramarine, kaolin (100 parts) should be heated overnight at 600 °C, if the clay is left for an extended period of time, over 3 hours in air, it should be reactivated with heating. The composition of china clay depends on the geographical location of the mine from which it was extracted and it is important to use low purity, specifically low feldspar, kaolin [7]. High levels of feldspar in the clay produce a ‘redder’ shade of ultramarine. Ideally, the clay should be mixed immediately with sodium carbonate (100 parts), bitumen emulsion (or any ‘sticky’ carbon source) (12 parts) and sulphur (60 parts), consistent with the medium recipe reported by Zerr and Rubencamp [17] and the Tone II recipe by Riffault, Vergnaud, and Toussaint [13]. The mixture should be ground, either by hand or mechanically, to create a homogeneous powder that ensures that the formed colour is uniform. The powder should then be made into a compact pellet (or brick for larger scale production) as the compaction minimises sulphur sublimation and oxidation and creates a reduced environment within the pellet. The first calcinations should be carried out at 750°C for around four hours and, crucially, at this stage exposure to air should be limited. This is achieved by using a close fitting lid to allow liberation of H_2_SO_4_ and SO_2_ whilst minimising air ingress (a sealed lid would likely lead to a dangerous build up of pressure within the vessel). The second calcination step progresses after the sample has been allowed to cool to 500°C within the furnace and the lid has been removed. Additional sulphur (30 parts) should be added with care; ignition is to be expected and care should be taken to avoid inhalation of the gaseous sulphurous fumes, especially H_2_SO_4_, liberated at this time. A furnace temperature of 500°C should be maintained for 2 hours, whereupon the sample can be removed from the furnace and allowed to cool to room temperature. Once cooled, the pellet can be scraped free of any charred and blackened material from the surface and then ground with a pestle and mortar. The pellets can be ground to any desired extent, but larger particle sizes produce a more vivid pigment. A washing step can be included at this stage to remove any excess sulphides, although this was not necessary in the present work.
